# Proapoptotic activity of Ukrain is based on *Chelidonium majus *L. alkaloids and mediated via a mitochondrial death pathway

**DOI:** 10.1186/1471-2407-6-14

**Published:** 2006-01-17

**Authors:** Daniel Habermehl, Bernd Kammerer, René Handrick, Therese Eldh, Charlotte Gruber, Nils Cordes, Peter T Daniel, Ludwig Plasswilm, Michael Bamberg, Claus Belka, Verena Jendrossek

**Affiliations:** 1 Department of Radiation Oncology, University Hospital of Tuebingen, Hoppe-Seyler-Str. 3, D-72076 Tuebingen, Germany; 2 Institute of Pharmacology and Toxicology, Division of Clinical Pharmacology, University Hospital of Tuebingen, Otfried-Mueller-Str. 45, D-72076 Tuebingen, Germany; 3 University Hospital, Department of Radiation Oncology, Petersgraben 4, Ch-4031 Basel, Switzerland; 4 OncoRay – Radiation Research in Oncology, Medical Faculty Carl Gustav Carus, Technical University Dresden, Fetscherstrasse 74, D-01307 Dresden, Germany; 5 Department of Clinical and Molecular Oncology, University Medical Center Charité, Campus Buch, Humboldt University, Lindenbergerweg 80, D-13125 Berlin, Germany

## Abstract

**Background:**

The anticancer drug Ukrain (NSC-631570) which has been specified by the manufacturer as semisynthetic derivative of the *Chelidonium majus *L. alkaloid chelidonine and the alkylans thiotepa was reported to exert selective cytotoxic effects on human tumour cell lines *in vitro*. Few clinical trials suggest beneficial effects in the treatment of human cancer. Aim of the present study was to elucidate the importance of apoptosis induction for the antineoplastic activity of Ukrain, to define the molecular mechanism of its cytotoxic effects and to identify its active constituents by mass spectrometry.

**Methods:**

Apoptosis induction was analysed in a Jurkat T-lymphoma cell model by fluorescence microscopy (chromatin condensation and nuclear fragmentation), flow cytometry (cellular shrinkage, depolarisation of the mitochondrial membrane potential, caspase-activation) and Western blot analysis (caspase-activation). Composition of Ukrain was analysed by mass spectrometry and LC-MS coupling.

**Results:**

Ukrain turned out to be a potent inducer of apoptosis. Mechanistic analyses revealed that Ukrain induced depolarisation of the mitochondrial membrane potential and activation of caspases. Lack of caspase-8, expression of cFLIP-L and resistance to death receptor ligand-induced apoptosis failed to inhibit Ukrain-induced apoptosis while lack of FADD caused a delay but not abrogation of Ukrain-induced apoptosis pointing to a death receptor independent signalling pathway. In contrast, the broad spectrum caspase-inhibitor zVAD-fmk blocked Ukrain-induced cell death. Moreover, over-expression of Bcl-2 or Bcl-x_L _and expression of dominant negative caspase-9 partially reduced Ukrain-induced apoptosis pointing to Bcl-2 controlled mitochondrial signalling events.

However, mass spectrometric analysis of Ukrain failed to detect the suggested trimeric chelidonine thiophosphortriamide or putative dimeric or monomeric chelidonine thiophosphortriamide intermediates from chemical synthesis. Instead, the *Chelidonium majus *L. alkaloids chelidonine, sanguinarine, chelerythrine, protopine and allocryptopine were identified as major components of Ukrain.

Apart from sanguinarine and chelerythrine, chelidonine turned out to be a potent inducer of apoptosis triggering cell death at concentrations of 0.001 mM, while protopine and allocryptopine were less effective. Similar to Ukrain, apoptosis signalling of chelidonine involved Bcl-2 controlled mitochondrial alterations and caspase-activation.

**Conclusion:**

The potent proapoptotic effects of Ukrain are not due to the suggested "Ukrain-molecule" but to the cytotoxic efficacy of *Chelidonium majus *L. alkaloids including chelidonine.

## Background

**Figure 1:**
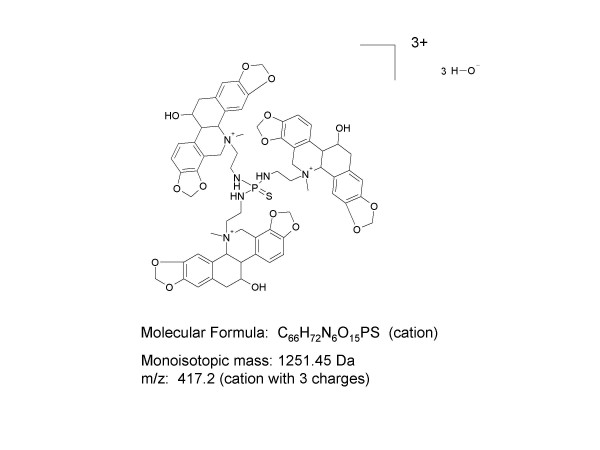
(a) Postulated trimeric structure of the "Ukrain-molecule".

The anticancer drug Ukrain has been specified by Nowicky Pharma (Vienna, Austria) as semi-synthetic derivative of thiotepa and the alkaloid chelidonine, a main component of the plant greater celandine (*Chelidonium majus *L., Papaveraceae) [1-3]. In the postulated molecular structure one central thiophosphortriamide molecule is surrounded by three molecules of chelidonine, which are bound in a covalent manner (Fig. 1).

During the last decades pre-clinical investigations pointed to promising antineoplastic activity of Ukrain. In these studies, Ukrain was suggested to exert selective cytotoxic effects on tumour cells without adverse side effects on normal cells and tissues [4]. Recently, Ukrain was also shown to inhibit tumour growth and metastasis of Lewis carcinoma in C57Bl6 mice [5]. Moreover, a recent report revealed radiosensitizing effects of Ukrain on human colorectal and glioblastoma cell lines *in vitro*, while normal human fibroblasts were protected against the cytotoxic effects of ionizing radiation [6]. However, the observations on selective cytotoxicity of Ukrain are still subject to controversial discussion [7]. In addition to the above mentioned promising pre-clinical data, some clinical investigations predominantly from Eastern Europe suggested beneficial effects of Ukrain in the treatment of patients suffering e.g. from bladder, breast, pancreatic, rectal or colorectal cancer when given as single drug or in combination with standard chemotherapeutic drugs or ionizing radiation (recently reviewed by [8]). However, the molecular mechanisms of Ukrain-induced antineoplastic effects are not yet completely understood. Apart from suggested immunomodulatory effects, induction of a growth arrest in the G2/M phase of the cell cycle and/or induction of apoptosis may be involved [7,9-13].

Apoptosis constitutes a highly regulated, physiological form of cell death involving complex intracellular proteolysis. In this scenario, specialized intracellular cysteine proteases known as caspases constitute central executioners of apoptosis that cleave a multitude of cellular substrates triggering morphological alterations and finally cell death [14]. There is accumulated evidence that caspases can either be activated by the extrinsic, death receptor dependent or the intrinsic, death receptor-independent mitochondrial pathway. Death receptor ligands, such as CD95 or TRAIL, trigger clustering of their respective receptors in the cytoplasmic membrane with recruitment of the adapter molecule FADD (Fas associated Death Domain) and pro-caspase-8 to form a multimeric death receptor-inducing complex (DISC). Proximity of several pro-caspase-8 molecules in the DISC allows autoproteolytic cleavage and thus activation of this initiator caspase with subsequent cleavage of downstream effector caspases such as caspase-3, -6 and -7 [15]. In contrast, application of cellular stress mostly triggers the so-called mitochondrial death pathway that critically involves disruption of the mitochondrial membrane potential with release of proapoptotic proteins including cytochrome c from the mitochondrial intermembrane space into the cytosol [16]. Release of cytochrome c culminates in the activation of pro-caspase-9 within a multimeric cytosolic death inducing complex, the apoptosome. Similar to caspase-8, the initiator caspase-9 triggers activation of the effector caspase-cascade that finally executes cell death [17-19]. A mitochondrial amplification loop mediated by caspase-8 triggered cleavage of the proapoptotic Bcl-2 protein Bid yielding truncated Bid (tBid) and tBid mediated release of cytochrome c from the mitochondrial intermembrane space constitutes a molecular link between the death receptor and the mitochondrial pathway that assures execution of apoptosis in cells with low initial caspase-8 cleavage upon death receptor stimulation [20]. Inversely, dependent on the cell line, complete activation of mitochondria and subsequent execution of drug-induced apoptosis may rely on a mitochondrial amplification loop mediated by caspase-3 and caspase-8 [21].

Induction of apoptosis is a common mechanism of the cytotoxic action of most DNA-damaging drugs and irradiation. However, tumour cells are often characterized by alterations in genes coding for proteins involved in apoptosis and survival signalling [22,23]. In this regard, increased levels of anti-apoptotic or decreased levels of pro-apoptotic proteins can cause acquired or intrinsic resistance against apoptosis induction by DNA-damaging antineoplastic drugs and ionizing radiation thereby contributing to treatment failure and poor clinical prognosis [24]. Thus, novel agents targeting aberrant apoptosis pathways or inducing alternative death pathways may be suited to overcome treatment resistance [16,25-34].

The putative proapoptotic and radiosensitizing effects together with its reported selective action on tumour cells make Ukrain an interesting novel agent for cancer treatment as single drug and in combination with radiation therapy. However, data regarding the cytotoxic activity of the drug are still conflicting and the molecular mechanisms of Ukrain-triggered cell death are not yet understood. Moreover, concerns about the chemical purity of this semisynthetic drug emerged [35].

Therefore, the aim of the present study was to characterize the apoptosis inducing capacity of Ukrain, to elucidate molecular mechanisms of Ukrain-induced apoptosis and to analyse the chemical identity of the drug preparation.

## Methods

### Chemicals and drugs

The pharmaceutical preparation "Ukrain" (NSC-631570) with an indicated concentration of 1 mg/ml "Ukrain-molecule") was obtained from Nowicky Pharmaceuticals (Vienna, Austria). Chelidonine, protopine, allocryptopine, chelerythrine, sanguinarine and thiotepa were purchased from Sigma-Aldrich (Munich, Germany). For *in vitro *investigations the alkaloids were dissolved in water (allocryptopine, chelerythrine and protopine), DMSO (chelidonine) or methanol (sanguinarine) as 1 mM, 10 mM or 100 mM stock solutions.

A native extract of greater celandine (*Chelidonium majus *L.) was obtained from Caelo & Loretz GmbH (Hilden, Germany). As indicated by the manufacturer the extract solution contained 0.35% (w/w) alkaloids as calculated from the content of chelidonine as major alkaloid component. For experimental use, the stock solution was adjusted to 1 mM chelidonine.

Etoposide and the pan-caspase-inhibitor zVAD-fmk were purchased from Alexis Biochemicals (San Diego, USA) and dissolved in DMSO. TMRE was obtained from Molecular Probes (Mobitec, Goettingen, Germany) and dissolved as a 25 μM stock solution in water. Hoechst 33342 and propidium iodide were purchased from Calbiochem (Bad Soden, Germany) and from Sigma (Munich, Germany), respectively, and dissolved in distilled water. Trypan Blue was from Sigma (Munich, Germany) and used as 0.04% solution for cytotoxicity assays.

Rabbit anti-cleaved caspase-3, rabbit anti-Bad, rabbit anti-PARP- and rabbit anti-cleaved PARP antibodies were obtained from Cell Signaling (Frankfurt, Germany). Anti-caspase-9-antibody and polyclonal rabbit anti-Bak-NT were purchased from Upstate (Biomol, Hamburg, Germany). Monoclonal mouse anti-caspase-8-mouse-antibody directed against the p18 subunit was obtained from BioCheck (Münster, Germany)[19]. Monoclonal mouse anti-Bcl-2 mouse-antibody was purchased from Santa Cruz Biotech (Heidelberg, Germany). Polyclonal rabbit anti-Bim was purchased from Pharmingen (Becton Dickinson, Heidelberg, Germany) and rabbit anti-Bid from R & D Systems (Wiesbaden, Germany). Horse-radish peroxidase-conjugated secondary antibodies were purchased from Santa-Cruz-Biotech (Heidelberg, Germany; anti-rabbit and anti-mouse) and Amersham-Pharmacia Biotech (Freiburg, Germany; anti-sheep), respectively. All other chemicals were obtained from Sigma (Munich, Germany) if not otherwise specified.

### Cell culture and cellular treatment

Jurkat A3 T-lymphoma cells were from American Type Culture Collection (Manassas, VA, USA). Caspase-9 DN expressing Jurkat cells, caspase-8 and FADD-negative Jurkat cells, CD95/TRAIL-resistant Jurkat A3 as well as Bcl-2 overexpressing Jurkat cells and the respective mock transfected control cells (Jurkat Vector) were used as already described [19,36,37]. Jurkat J16 control cells as well as cFLIP-L expressing Jurkat cells were kindly provided by J. Tschopp, Epalinges, Switzerland [38]. Jurkat cells were grown in RPMI 1640 medium supplemented with 10% fetal calf serum (Invitrogen-Gibco, Karlsruhe, Germany) and 100 U/ml penicilline and 100 μg/ml streptomycine (Invitrogen-Gibco, Karlsruhe, Germany) and maintained in a humidified incubator at 37°C and 5% CO_2_.

Irradiation was performed with 6 MV photons from a Siemens Mevatron linear accelerator with a dose rate of 4 Gy per min at room temperature.

### Cytotoxicity assay

Cytotoxicity of Ukrain was measured by standard live-dead staining. To this end, cells were incubated for 10 min with Trypan Blue staining solution (0.04%). The number of viable (transparent) and dead (blue) cells was determined by light microscopic analysis.

### Determination of apoptosis

Cell death was analysed by fluorescence microscopy upon combined staining of the cells with Hoechst 33342 and propidium iodide. In brief, cells were incubated for 15 min with Hoechst 33342 (1.5 μM) or with Hoechst 33342 (1.5 μM) and propidium iodide (2.5 μg/ml) where indicated. Cell morphology was then determined by fluorescence microscopy (Zeiss Axiovert 200, Carl Zeiss, Jena, Germany) using an excitation wavelength filter of 380 nm. Cells were analysed with 40fold magnification and documented using a CCD camera device (Zeiss Axiocam MRm). At least 250 cells were counted for each value.

Moreover, cell death was quantified by flow cytometry using light scatter characteristics employing a FACS Calibur flow cytometer (Becton Dickinson, Heidelberg, Germany).

### Determination of mitochondrial transmembrane potential

The mitochondrial transmembrane potential (Δψm) was analysed by flow cytometry using the Δψm-specific stain TMRE (tetramethylrhodamine ethylester perchlorate) (Molecular Probes, Mobitech, Göttingen Germany). To this end, cells were loaded for 30 min at 37°C with 25 nM TMRE and subsequently analysed by flow cytometry. Preincubation with 1 μM of the proton ionophore CCCP (carbonylcyanide-m-chlorophenylhydrazone) was used as a positive control for complete depolarisation.

### Determination of caspase-activation

Caspase activation was determined by Western blot analysis of cytosolic extracts upon treatment with Ukrain or defined alkaloid preparations. To this end, cells were resuspended in 3fold concentrated lysis buffer (final concentration: 62.5 mM Tris-HCl (pH = 6.8), 2% (w/v) SDS, 10% (v/v) Glycerol, 50 mM DDT, 0.01% (w/v) bromphenol-blue in PBS) and subsequently boiled for 10 min at 100°C.

Ten μl lysate (corresponding to 10^5 ^cells) were separated by SDS-PAGE and subsequently blotted onto PVDF-membranes (Pharmacia, Freiburg, Germany). Equal protein loading was confirmed by Coomassie Blue staining.

Blots were blocked for 1 h in TBS buffer containing 0.05% Tween 20 and 5% non fat dried milk (3% non fat dried milk in case of caspase-9 antibody). The membrane was then incubated over night at 4°C with the respective primary antibodies. After repeated washings with PBS/Tween-20 (0.05%) the membrane was incubated with the respective secondary antibody (1:10 000) in TBS/Tween for 1 h at room temperature and again washed several times with TBS/Tween. The detection of antibody binding was performed by enhanced chemoluminescence staining (ECL Western blotting analysis system, Amersham-Pharmacia Biotech, Freiburg, Germany).

In addition, caspase activation was quantified using the CaspACE^® ^FITC-VAD-fmk in situ marker which consists of a FITC-conjugate of the cell permeable pan-caspase-inhibitor VAD-fmk (Promega, Mannheim, Germany). Within the cell the inhibitor binds to activated caspases and serves as *in situ *marker for caspase-activation. CaspACE^®^-staining was performed according to the manufacturer's guidelines.

### Chromatographic separation and mass spectrometric investigations of *Chelidonium majus *extract and the pharmaceutical preparation Ukrain (NSC-631570)

#### Dissolution of extract and Ukrain prior to analysis

Before analysis both the *Chelidonium majus *L. extract and Ukrain were diluted with methanol/water. Ten μl of *Chelidonium majus *extract were diluted with 500 μl methanol/water (90/10, v/v), stirred for 10 min, centrifuged and the brown supernatant was used for analyis. Twenty μl of Ukrain were diluted with 100 μl methanol/water (90/10, v/v), and the light green solution was used for further investigations. In each case 10 μl of the particle free methanolic/water solutions were injected into the HPLC system coupled to ESI-MS interface.

#### High-performance-liquid-chromatography

The HPLC system consisted of a Surveyor quaternary pump (Thermo Finnigan; San Jose, California; USA), Surveyor PDA detector (UV-range scanned: 200–800 nm in 5 nm intervals) and Surveyor autosampler. Reversed-phase chromatography was carried out on a Grom Saphir 110 C8, (3 μm, 150 × 2 mm) column (Grom Analytik; Rottenburg, Germany). The mobile phase consisted of water + 0.1% formic acid (eluent A) and acetonitrile + 0.1% formic acid (eluent B); flow rate was 200 μl/min. Formic acid was necessary for improving peak shape. The gradient was applied as follows: 0 min: 100% A – 5 min: 100% A – 1 min: 95% A – 50 min: 50% A – 55 min: 50% A – 56 min: 100% A. The column was washed with pure methanol and pure acetonitrile after each run for 15 min and equilibrated with 100% A for 3 min.

#### Electrospray and MS settings

All mass spectra were obtained using a Thermo Finnigan (San Jose, California; USA) TSQ Quantum triple quadrupole mass spectrometer equipped with an electrospray interface coupled to the Surveyor HPLC-system and operated in positive ionisation mode. For data processing the Xcalibur 1.3 software was used. The ESI-interface was locked in a 90° position towards the orifice and operated at 3.8 kV. Capillary temperature and sheath gas were set to 320°C and 30 (arbitrary) units, respectively. Auxillary gas was set to 12 (arbitrary) units to enhance signal intensity.

##### Full scan MS scans

Conventional (non MS/MS) full scans in centroid mode using the third quadrupole as mass selector (Q3 full scan) were recorded in the range of m/z 100–1000 within a dwell time of 0.7 s. Source CID (collision induced fragmentation) voltage was 10 V, which minimizes analyte-solvent adducts.

##### MS/MS product ion scans

Within one LC-MS run up to four product ion scans for different precursor masses were combined. Source CID voltage was set to constant value of 10 V and collision energy to 35 V (for 354.1 m/z and 370.1 m/z) and to 40 V (for 332.1 m/z and 348.2 m/z), respectively and the argon collision gas was maintained at a pressure of 1.3· 10^-6 ^bar. Product ion spectra were recorded with a scan time of 0.5 s for each mass, a peak width of 0.7 for Q1 and Q3 and within a range of m/z 40 to m/z of the moleclular ion + 10 mass units.

### Mass spectrometric fragmentation behaviour of reference compounds

For each reference compound (Fig. 10), a solution with an approximate concentration of 0.1 mg/ml was prepared by dissolving the dry substances in methanol. Ten μl of these standard solutions were injected separately and analysed by LC-MS and LC-MS/MS under the same conditions as the *Chelidonium majus *extract and Ukrain. The data were then evaluated with respect to ionisation efficiency, compound stability in the electrospray source and adduct formation.

### Statistical analysis

Experiments were at least performed in triplicates. Data were represented as means ± SD. Where appropriate, one-way ANOVA test or paired t-test were performed using GraphPad InStat version 3.00 for Windows 95, GraphPad Software, San Diego California USA, .

## Results

### Ukrain induces apoptosis in Jurkat T-lymphoma cells

**Figure 2:**
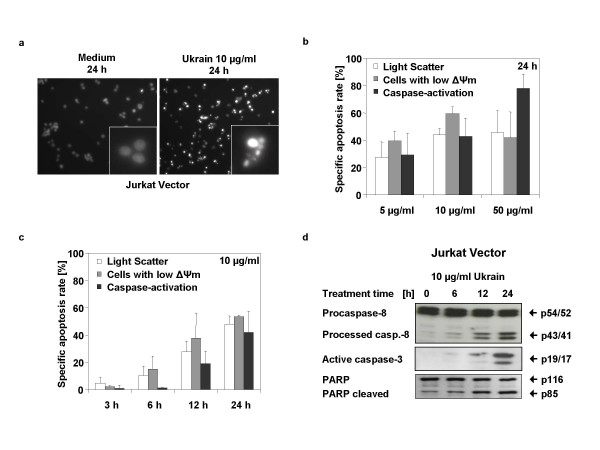
**(a) Ukrain induces morphological alterations indicative for apoptosis. **Mock-transfected Jurkat control cells (Jurkat Vector) were treated for 24 h with medium or 10 μg/ml Ukrain and subsequently stained with Hoechst33342. Apoptotic cells show characteristic chromatin condensation and nuclear fragmentation. Micrographs of a representative experiment are shown. **(b) Ukrain induces apoptosis in a concentration-dependent manner**. Jurkat Vector cells were treated for 24 h with medium or 5, 10 and 50 μg/ml Ukrain. Apoptosis was then quantified by flow cytometric analysis using light scatter characteristics (white bars), determination of the mitochondrial membrane potential Δψm upon staining with the potential sensitive dye TMRE (grey bars) and detection of caspase-activation upon staining with CaspACE^® ^(black bars). Data show specific apoptosis (% apoptosis in Ukrain-treated cells minus % apoptosis in untreated control cells) as means ± SD (n ≥ 3). **(c) Ukrain induces apoptosis in a time-dependent manner**. Jurkat Vector cells were treated with medium or 10 μg/ml Ukrain for 3, 6, 12 and 24 h and subsequently analysed by flow cytometry using light scatter characteristics (white bars), determination of the mitochondrial membrane potential upon staining with the potential sensitive dye TMRE (grey bars) and detection of caspase-activation upon staining with CaspACE^® ^(black bars). Data represent specific apoptosis (means ± SD, n ≥ 3). **(d) Ukrain induces activation of caspases**. Jurkat Vector cells were treated with medium or 10 μg/ml Ukrain for 6, 12 and 24 h. Caspase activation was determined by Western blot analysis of cytosolic extracts with specific antibodies against caspase-8 and caspase-3 and their respective cleavage products. In addition, caspase-3 activation was verified by detection of the cleavage of the caspase-3 substrate PARP. Data from one representative experiment are shown.

**Figure 3:**
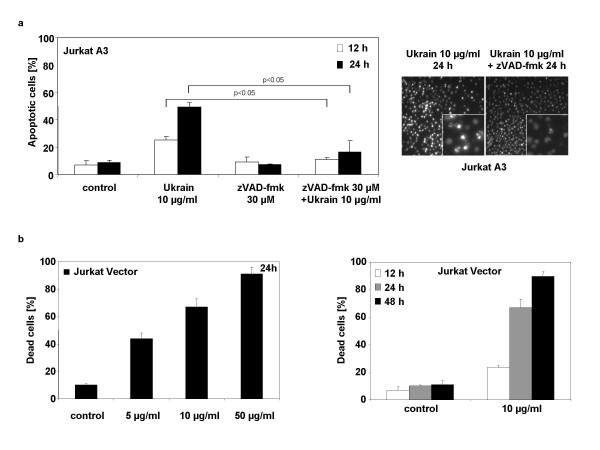
**(a) Ukrain-induced apoptosis depends on caspase-activation**. Jurkat A3 cells were pre-incubated for 2 h with medium supplemented with 30 μM of the pan-caspase-inhibitor zVAD-fmk or left untreated. Subsequently, cells were treated for 24 h with medium or 10 μg/ml Ukrain. Apoptosis was then quantified by counting of the cells with apoptotic nuclear morphology (left panel). Data show means ± SD, n = 3. Paired t-test was performed using GraphPad InStat version 3.00 for Windows 95, GraphPad Software, San Diego California USA, . Micrographs showing reduced induction of apoptotic nuclear morphology upon combined treatment are depicted in the right panel. **(b) Ukrain exerts concentration- and time-dependent cytotoxic effects on Jurkat T-lymphoma cells**. Jurkat Vector cells were treated for 24 h with medium, 5, 10 and 50 μg/ml Ukrain (left panel) or for 0, 12, 24 or 48 h with 10 μg/ml Ukrain (right panel). The number of living and dead cells was then quantified by light microscopy using Trypan Blue Exclusion. Data show means ± SD, n = 3.

Earlier reports suggested an involvement of apoptosis in the antineoplastic activity of Ukrain [9,10,12,13]. Therefore, in a first set of experiments we analysed the sensitivity of Jurkat Vector and A3 cells to apoptosis induction by Ukrain. Evaluation of nuclear morphology by fluorescence microscopy upon treatment with increasing drug-concentrations revealed Ukrain-triggered morphological alterations that are indicative for apoptotic cell death including chromatin condensation, nuclear shrinkage and fragmentation (Fig. 2a). In line with this observation, Ukrain induced a concentration- (Fig. 2b, white bars) and time-dependent (Fig. 2c, white bars) increase of apoptotic rates in Jurkat Vector cells compared to the respective untreated controls as determined by flow cytometry using light scatter characteristics indicative for cellular shrinkage. Interestingly, Ukrain also induced a concentration-dependent breakdown of the mitochondrial membrane potential (Fig. 2b, grey bars). Similar to the results regarding cellular shrinkage, Ukrain-induced alterations of mitochondrial function were observed within 6 h after treatment with 10 μg/ml Ukrain (Fig. 2c, grey bars). Moreover, Ukrain-treatment triggered a time- and concentration-dependent activation of caspases as indicated by flow cytometric detection of increased rates of CaspACE^®^-fluorescence (Fig 2b+c, black bars). These data were corroborated by detection of cleavage of caspase-3 and -8 as well as of the caspase-3 substrate PARP that was observed within 12 h after treatment with 10 μg/ml Ukrain (Fig. 2d). To evaluate importance of caspase-activation in Ukrain-triggered apoptosis, apoptosis rates were determined in the presence of the broad-spectrum caspase-inhibitor zVAD-fmk. Inhibition of caspase-activation by pre-incubation with zVAD-fmk resulted in a reduced amount of Ukrain-induced apoptosis compared to cells treated with Ukrain in the absence of zVAD-fmk (Fig. 3a). In addition, to prove cytotoxic efficacy of Ukrain in the Jurkat T-lymphoma cell model, the number of living and dead cells was determined by Trypan Blue Exclusion. As shown in Fig. 3b, Ukrain-treatment increased the percentage of dead cells in a time- and concentration-dependent manner.

### Signalling pathways of Ukrain-induced apoptosis

Up to now our data indicated that Ukrain triggers activation of caspases and alteration of mitochondrial function. To gain further insight into the molecular requirements for Ukrain-induced apoptosis we subsequently analysed the contribution of death receptor-dependent and mitochondrial apoptosis signalling events.

**Figure 4:**
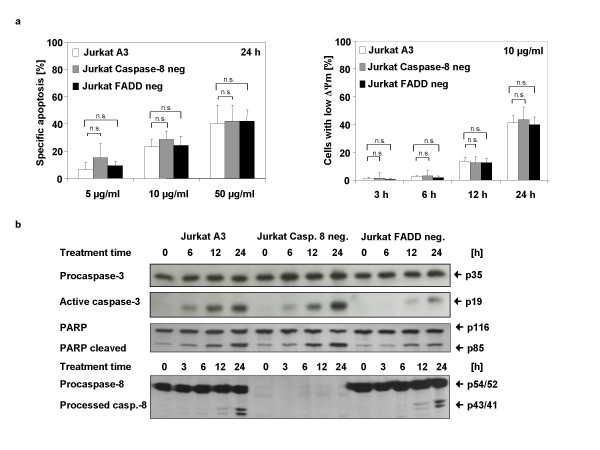
**(a) Expression of caspase-8 and FADD are not essential for Ukrain-induced apoptosis**. Jurkat A3 control cells as well as FADD-deficient and caspase-8-deficient Jurkat cells were treated for 24 h with medium or 5, 10 and 50 μg/ml Ukrain. Apoptosis was then determined by flow cytometry using light scatter characteristics (left panel). Alternatively, A3, FADD-deficient and caspase-8-deficient Jurkat cells were treated for 3, 6, 12 and 24 h with medium or 10 μg/ml Ukrain. Apoptosis induction was then quantified by determination of the mitochondrial membrane potential (Δψm) (right panel). Data show induction of specific apoptosis (means ± SD; n ≥ 3). One-way ANOVA was performed using GraphPad InStat version 3.00 for Windows 95, GraphPad Software, San Diego California USA, . **(b) Expression of caspase-8 and FADD are not essential for Ukrain-induced caspase-activation**. Jurkat A3 as well as FADD-deficient and caspase-8-deficient Jurkat cells were treated for 6, 12 and 24 h with medium or 10 μg/ml Ukrain. Caspase activation was then determined by Western blot analysis of cytosolic extracts with specific antibodies against caspase-3, cleaved caspase-3, PARP and cleaved PARP, respectively. In addition, cells were treated for 3, 6, 12 and 24 h with medium or 10 μg/ml Ukrain and Western blot analysis with specific antibodies against caspase-8 was performed. Data from one representative experiment are shown.

**Figure 5:**
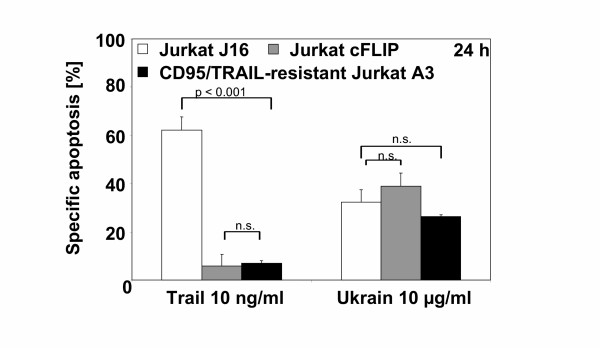
**Expression of cFLIP-L or resistance to death receptor ligands do not interfere with Ukrain-induced apoptosis **Jurkat J16 control cells, cFLIP-L expressing Jurkat cells and CD95/TRAIL resistant Jurkat cells were treated for 24 h with medium, 10 ng/ml TRAIL or 10 μg/ml Ukrain. Apoptosis was then quantified by fluorescence microscopy upon Hoechst33342-staining and counting of cells with apoptotic morphology (means ± SD; n = 3). One-way ANOVA was performed using GraphPad InStat version 3.00 for Windows 95, GraphPad Software, San Diego California USA, .

Since our results revealed activation of caspase-8 upon Ukrain-treatment, the importance of the death receptor pathway was analysed using Jurkat cells lacking caspase-8 or the adapter protein FADD, Jurkat cells expressing the caspase-8 inhibitor cFLIP-L as well as the respective control cells (Fig. 4 and Fig. 5) [38]. In addition, variant Jurkat A3 cells resistant to CD95 and TRAIL-induced apoptosis (CD95/TRAIL-resistant Jurkat cells) were used (Fig. 5) [36]. Lack of FADD and caspase-8 as well as expression of cFLIP-L were verified by Western blot analysis (data not shown). Resistance of CD95/TRAIL-resistant Jurkat cells to death receptor-induced apoptosis was verified by fluorescence microscopy upon stimulation with 10 ng/ml TRAIL (Fig. 5).

Ukrain induced mitochondrial damage and apoptosis in caspase-8- and FADD-negative cells in a concentration- and time-dependent manner to the same extent as in Jurkat A3 control cells (Fig. 4a right and left panel, respectively). Moreover, activation of caspases was clearly detectable in Ukrain-treated caspase-8- and FADD-deficient cells although a slight delay in the activation of caspase-3 and cleavage of the caspase-3 substrate PARP was observed in the FADD-deficient cells (Fig. 4b). Consistent with these findings, cFLIP-L expressing Jurkat cells as well as CD95/TRAIL resistant Jurkat cells that failed to undergo apoptosis upon treatment with 10 ng/ml TRAIL were sensitive to Ukrain-induced apoptosis to the same extent as the control cells (Fig. 5).

These data reveal that lack of caspase-8 or FADD, two essential components of the death receptor pathway, expression of cFLIP-L or resistance to death receptor ligands do not abrogate apoptosis upon Ukrain-treatment. In addtion, Ukrain-treatment induced mitochondrial alterations. Therefore, we subsequently tested the hypothesis that similar to DNA-damaging anticancer drugs, Ukrain may trigger apoptosis via the mitochondrial apoptosis signalling cascade. Mitochondrial death pathways are characterized by early depolarisation of the mitochondrial membrane potential and activation of caspases downstream of the mitochondria. These events are regulated by pro- and anti-apoptotic Bcl-2-like proteins [39]. In this context, anti-apoptotic Bcl-2 and Bcl-x_L _function as guardians of mitochondrial integrity preventing mitochondrial damage and caspase-activation triggered by pro-apoptotic Bcl-2 family members e.g. Bax and Bak at the level of the mitochondria [40].

**Figure 6:**
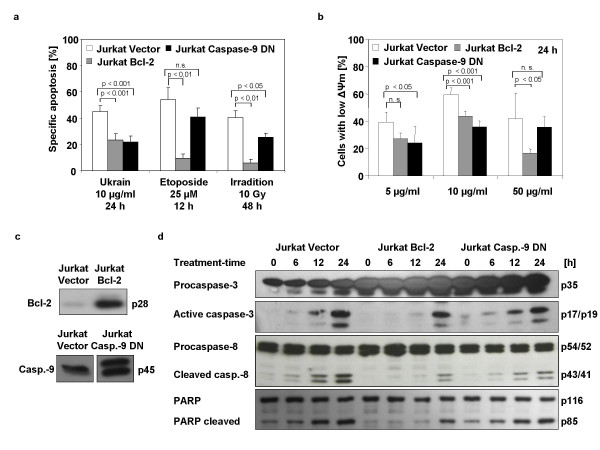
**(a) Overexpression of Bcl-2 and expression of a dominant negative caspase-9 partially reduce Ukrain-induced apoptosis**. Jurkat Vector cells, Jurkat Bcl-2 cells as well as Jurkat Caspase-9 DN cells were treated for 24 h with medium or 10 μg/ml Ukrain, for 12 h with medium or 25 μM Etoposide and for 48 h with medium or irradiated with 10 Gy 48 h prior to determination of apoptosis. Apoptosis was quantified by fluorescence microscopy upon Hoechst33342-staining by counting cells with apoptotic nuclear morphology. Data show specific apoptosis (apoptosis rates of treated cells minus apoptosis rates of untreated cells) as means ± SD (n = 3). One-way ANOVA was performed using GraphPad InStat version 3.00 for Windows 95, GraphPad Software, San Diego California USA, . **(b) Overexpression of Bcl-2 partially inhibits Ukrain-induced mitochondrial damage**. Jurkat Vector, Jurkat Bcl-2 as well as Jurkat Caspase-9 DN cells were treated for 24 h with medium or 5, 10 and 50 μg/ml Ukrain. Apoptosis induction was then quantified by determination of the mitochondrial membrane potential (Δψm) (right panel). Data show induction of specific apoptosis (means ± SD; n ≥ 3). One-way ANOVA was performed using GraphPad InStat version 3.00 for Windows 95, GraphPad Software, San Diego California USA, . **(c) Verification of variant Jurkat clones**. Expression of Bcl-2 in Jurkat Vector and Bcl-2 overexpressing Jurkat cells (Jurkat Bcl-2) (upper panel) as well as expression of caspase-9 in Jurkat Vector and Jurkat cells expressing a dominant negative caspase-9 (Jurkat Caspase-9 DN) (lower panel) were verified by Western blot analysis of cytotsolic extracts with the respective antibodies. **(d) Overexpression of Bcl-2 and expression of a dominant negative caspase-9 only slightly inhibit Ukrain-induced caspase-activation. **Jurkat Vector cells, Jurkat Bcl-2 cells as well as Jurkat Caspase-9 DN cells were treated for 0, 6, 12 and 24 h with medium or 10 μg/ml Ukrain. Caspase-activation was then determined by Western blot analysis of cytosolic extracts with antibodies against full length and active caspase-3, caspase-8 as well as PARP and cleaved PARP. Data from one representative experiment are shown.

To analyse the contribution of the mitochondrial death pathway Ukrain-induced apoptosis was determined in Jurkat cells overexpressing anti-apoptotic Bcl-2, Bcl-x_L _or a dominant negative caspase-9, three known antagonists of the mitochondrial death pathway, as well as the respective mock-transfected control cells (Jurkat Vector). Expression of Bcl-2, Bcl-x_L _and caspase-9 were controlled by Western blot analysis (Fig. 6c and data not shown).

**Figure 7:**
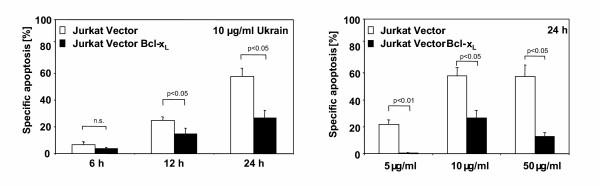
**Overexpression of Bclx_L _reduces Ukrain induced apoptosis**. Jurkat Vector cells and Jurkat cells overexpressing anti-apoptotic Bcl-x_L _(Jurkat Bcl-x_L_) were treated with 10 μg/ml Ukrain for 6, 12 and 24 h (left panel) or for 24 h with 5, 10 and 50 μg/ml Ukrain (right panel). Apoptosis was then measured by fluorescence microscopy upon Hoechst33342-staining. Data show means ± SD (n = 3). Paired t-test was performed using GraphPad InStat version 3.00 for Windows 95, GraphPad Software, San Diego California USA, ).

In our hands, overexpression of Bcl-2 only partially inhibited apoptosis induction upon treatment with Ukrain as determined by fluorescence microscopy upon Hoechst 33342 staining (Fig. 6a). Similar results were obtained in cells overexpressing Bcl-x_L _(Fig. 7). Moreover, Bcl-2 almost completely abrogated apoptosis induction upon treatment with etoposide or ionizing radiation, two paradigmatic stimuli of the mitochondrial death pathway. Expression of a dominant negative caspase-9 mutant inhibited Ukrain-induced apoptosis to a similar extent, while apoptosis induction upon treatment with etoposide or ionizing radiation was less affected (Fig. 6a).

In line with these findings, overexpression of Bcl-2 reduced Ukrain-induced mitochondrial damage while expression of the dominant negative caspase-9 was less effective, at least at increased drug concentrations (Fig. 6b). The moderate effects on apoptosis induction observed by flow cytometric analysis were reflected by determination of caspase-activation using Western blot analysis that revealed reduction but not inhibition of caspase-activation and PARP-cleavage in Jurkat Bcl-2 cells compared to control cells (Fig. 6d). In this regard, the small amount of PARP-cleavage observed in untreated Jurkat Vector and Jurkat Caspase-9 DN cells was attributed to increased levels of spontaneous apoptosis in untreated cells compared to untreated Bcl-2 control cells.

Since expression of Bcl-2, Bcl-x_L _and the dominant negative caspase-9 reduced Ukrain-induced apoptosis pointing to the contribution of mitochondrial signalling events we subsequently analysed whether Ukrain-treatment would affect pro-apoptotic members of the Bcl-2 family. However, treatment with Ukrain did not alter expression levels or integrity of the pro-apoptotic Bak, Bid or Bad (data not shown).

### Mass spectrometric analysis of Ukrain-components

**Figure 8:**
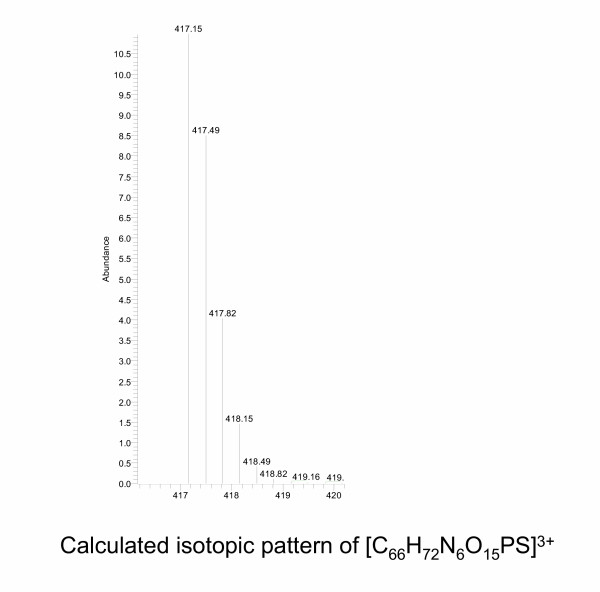
Calculated isotopic pattern of [C_66_H_72_N_6_O_15_PS]^3+^.

The main component of Ukrain as pharmaceutical preparation has been suggested to consist of three chelidonine moieties crosslinked with one thiothepa unit in the center representing the "Ukrain molecule" [1-3]. The postulated chemical structure of the "Ukrain-molecule" (NSC-631570) is depicted in Fig. 1 indicating its cationic structure with an calculated isotopic pattern for [C_66_H_72_N_6_O_15_PS]^3+ ^as given in Fig. 8.

**Figure 9:**
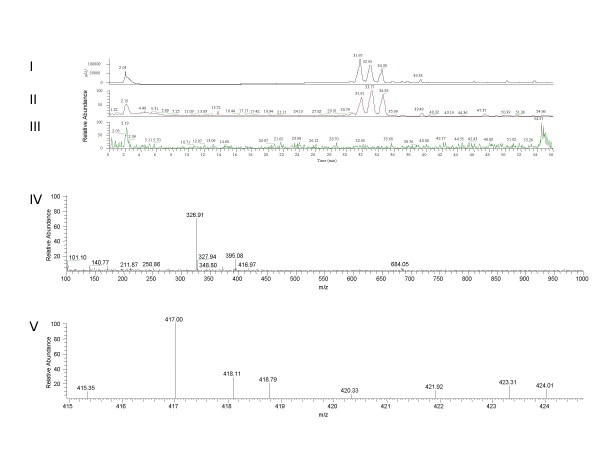
**LC-UV-MS and LC-UV-MS/MS spectra of Ukrain^®^. Extracted ion chromatogram for m/z 417.2. I**: LC-UV (290 nm) chromatogram; **II**: Total ion current (TIC) of mass range m/z 100 to 1000; **III**: LC-MS ion chromatogram of m/z 417.2 (extracted ion chromatogram); **IV**: Mass spectrum of peak with retention time 54.6 min; **V**: Zoom of mass range m/z 415 to 425 showing isotopic pattern of m/z 417.

To check integrity of the "Ukrain-molecule", mass spectrometric analysis of this pharmaceutical preparation was performed. Surprisingly, the extracted ion chromatogram (EIC) for m/z 417.2 (Fig. 9) demonstrated that the reported semisynthetic thiothepa derivative Ukrain was completely absent in the pharmaceutical preparation Ukrain. Moreover, no dimeric or monomeric condensation products of thiotepa with two or one chelidonine molecules were found (data not shown).

**Figure 10:**
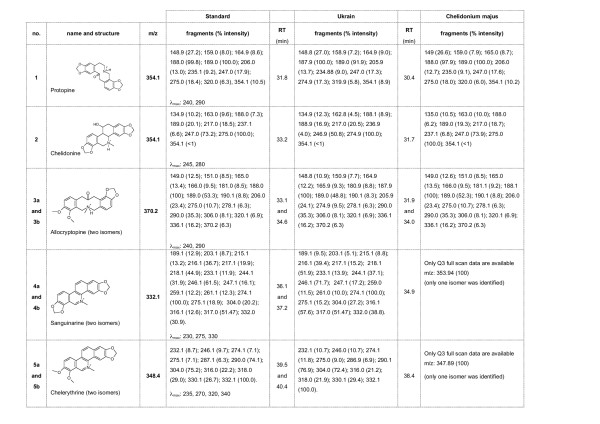
**Constituents of Ukrain and *Chelidonium majus *L. extract**: Comparison of MS/MS fragmentation with reference standards.

**Figure 11:**
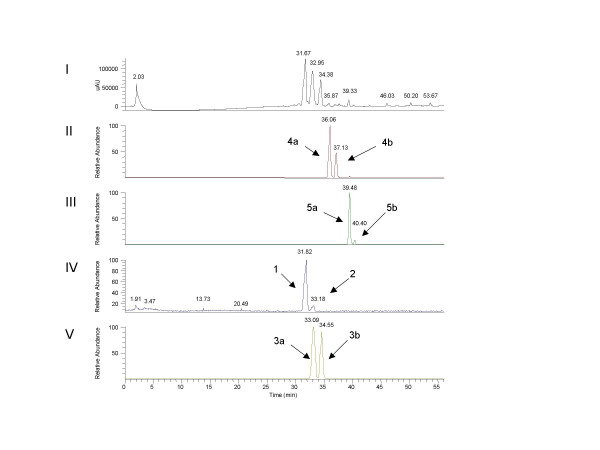
**LC-UV-MS and LC-UV-MS/MS spectra of Ukrain**. **I**: LC-UV (290 nm) chromatogram; **II**: LC-MS/MS ion chromatogram of m/z 332.1; **III**: LC-MS/MS ion chromatogram of m/z 348.4; **IV**: LC-MS/MS ion chromatogram of m/z 354.1; **V**: LC-MS/MS ion chromatogram of m/z 370.2.

Instead, mass spectrometric analysis of the pharmaceutical preparation Ukrain revealed five major components of the preparation including the *Chelidonium majus *alkaloids protopine (peak no 1, Fig. 10 and Fig. 11) chelidonine (peak no 2, Fig. 10 and Fig. 11), allocryptopine (peak no 3a and no 3b, Fig. 10 and Fig. 11), sanguinarine (peak no 4a and 4b, Fig. 10 and Fig. 11) and chelerythrine (peak no 5a and 5b, Fig. 10 and Fig. 11). Identity of the alkaloids was shown by the detection of molecules with identical MS/MS-fragmentation pattern as the reference compounds (Fig. 10).

**Figure 12:**
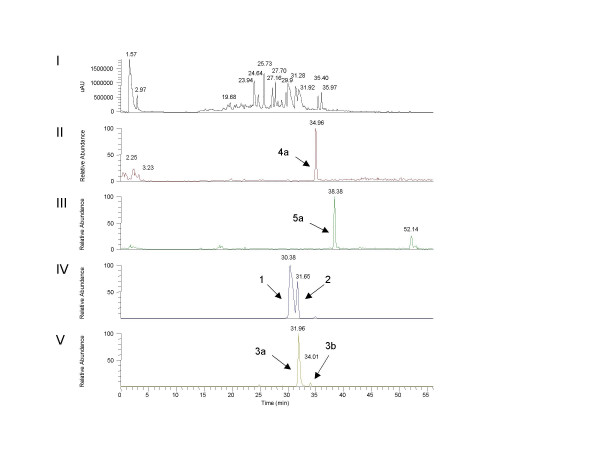
**LC-UV-MS and LC-UV-MS/MS spectra of *Chelidonium majus *L. extract I**: LC-UV (290 nm) chromatogram; **II**: LC-MS ion chromatogram of m/z 332.1 (extracted ion chromatogram); **III**: LC-MS ion chromatogram of m/z 348.4 (extracted ion chromatogram); **IV**: LC-MS/MS ion chromatogram of m/z 354.1; **V**: LC-MS/MS ion chromatogram of m/z 370.2.

In addition, the same alkaloid components were identified in commercially available extracts of *Chelidonium majus *(see peaks no 1–5, Fig. 10 and Fig. 12).

### Extracts of *Chelidonium majus *extracts have similar proapoptotic potency as Ukrain

**Figure 13:**
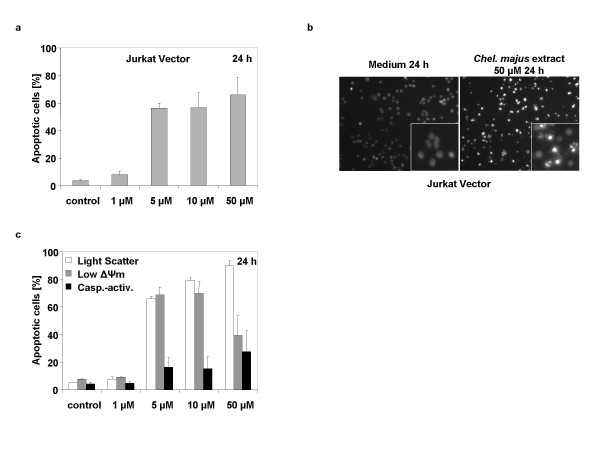
**(a) *Chelidonium majus *L. extracts induce apoptosis**. Jurkat Vector cells were treated for 24 h with medium supplemented with solvent or 1, 5, 10 and 50 μM *Chelidonium majus *L. extract adjusted to the respective chelidonine concentration. Apoptosis induction was analysed by fluorescence microscopy upon combined staining with Hoechst33342 and propidium iodide. The percentage of cells with characteristic apoptotic nuclear morphology was determined by counting a minimum of 250 cells per data point in each of at least three independent experiments. Data represent the percentage of cells with apoptotic nuclear morphology (means ± SD, n = 3). **(b) *Chelidonium majus *L. extracts induce chromatin condensation and nuclear fragmentation**. Jurkat Vector cells were treated for 24 h with medium supplemented with solvent or 50 μM *Chelidonium majus *extract adjusted to the respective chelidonine-concentration. Apoptosis induction was analysed by fluorescence microscopy upon combined staining with Hoechst33342 and propidium iodide. Characteristic micrographs of nuclear morphology are shown. **(c) *Chelidonium majus *L. extracts induce mitochondrial damage and caspase-activation**. Jurkat vector cells were treated for 24 h with medium supplemented with solvent or 1, 5, 10 and 50 μM *Chelidonium majus *L. extract adjusted to the respective chelidonine-concentration. Induction of apoptosis was then measured using flow cytometry (cell shrinkage: white bars; breakdown of mitochondrial membrane potential: grey bars; caspase-activation: black bars). Data show means ± SD (n ≥ 3).

Based on the finding that instead of the suggested trimeric chelidonine thiophosphortriamide the pharmaceutical preparation Ukrain contains a mixture of several known *Chelidonium majus *L alkaloids we wondered whether extracts of *Chelidonium majus *L. would also induce apoptosis in Jurkat T-lymphoma cells. To this end, we quantified apoptosis induction in Jurkat Vector cells upon treatment with a commercially available *Chelidonium majus *L. extract. As expected, *Chelidonium majus *L. extracts adjusted to 5 to 50 μM chelidonine (corresponding to 1.765 μg/ml and 17.65 μg/ml chelidonine, respectively) as major alkaloid compound induced massive apoptosis in Jurkat Vector cells within 24 h in a concentration-dependent manner as quantified by fluorescence microscopy upon staining with Hoechst 33342 (Fig. 13a). While a concentration of 1 μM was almost ineffective, 5 μM extract yielded massive apoptosis. A further increase in the alkaloid concentration led only to a slight increase in the number of apoptotic cells compared to the values obtained with 5 μM extract. The characteristic nuclear morphology observed upon treatment with *Chelidonium majus *L. extracts is depicted in Fig. 13b. Quantification of cell shrinkage and of depolarisation of the mitochondrial membrane potential gave similar results (Fig 13c white and grey bars, respectively). Interestingly, in contrast to Ukrain, treatment with *Chelidonium majus *L. extracts induced only low levels of caspase-activation (Fig. 13c, black bars).

### Chelidonine is a potent apoptosis inducing component of Ukrain

Up to now our data indicated that the pharmaceutical preparation Ukrain and *Chelidonium majus *extracts both constitute mixtures of diverse *Chelidonium majus *L. alkaloids and induce apoptosis of Jurkat T-lymphoma cells with almost similar potency. Consequently, we next aimed to define the apoptosis inducing alkaloids among the components of the Ukrain-preparation identified by mass spectrometry.

**Figure 14:**
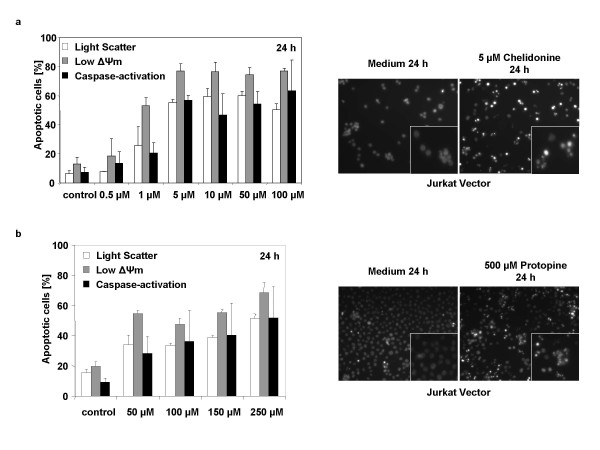
**(a) Chelidonine potently induces apoptosis in Jurkat Vector cells**. Jurkat Vector cells were treated for 24 h with medium supplemented with solvent or 1, 5, 10, 50 and 100 μM chelidonine. Apoptosis was quantified by flow cytometric analysis using light scatter characteristics (white bars), measurement of mitochondrial membrane potential Δψm (grey bars) and the activation of caspases (black bars) (left panel). Data show means ± SD (n ≥ 3). Fluorescence microscopy of Hoechst33342 stained cells treated for 24 h with 5 μM chelidonine revealed massive chromatin condensation and nuclear fragmentation (right panel). **(b) Protopine induces apoptosis in Jurkat Vector cells at increased drug concentrations**. Jurkat Vector cells were treated for 24 h with medium or 50, 100, 150 and 250 μM protopine. Protopine-induced apoptosis was then quantified by flow cytometric analysis using light scatter characteristics (white bars), measurement of mitochondrial membrane potential Δψm (grey bars) and caspase- activation (black bars) (left panel). Data show means ± SD (n ≥ 3). Fluorescence microscopy upon combined staining with Hoechst33342 and propidium iodide treated for 24 h with 500 μM protopine showed increased levels of cells undergoing apoptosis (right panel).

**Figure 15:**
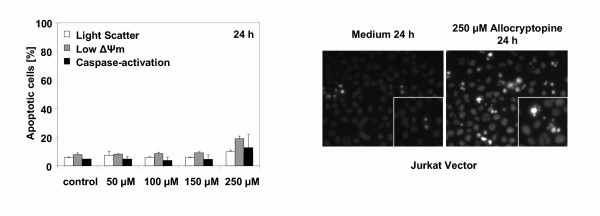
**Allocryptopine fails to induce apoptosis at concentrations below 250 μM**. Allocryptopine-induced apoptosis was quantified by flow cytometric analysis using light scatter characteristics (white bars), measurement of mitochondrial membrane potential Δψm (grey bars) and the activation of caspase-3 (black bars) upon treatment of Jurkat Vector cells for 24 h with medium or 0, 50, 100, 150 and 250 μM allocryptopine. Data show means ± SD (n ≥ 3) (left panel). Fluorescence microscopy upon combined staining with Hoechst33342 and propidium iodide staining of cells treated with 250 μM allocryptopine for 24 h showed increased numbers of cells with apoptotic morphology upon treatment (right panel).

To this end apoptosis induction by chelidonine, protopine, allocryptopine, sanguinarine and chelerythrine was analysed in Jurkat T-lymphoma cells. In these investigations chelidonine, sanguinarine and chelerythrine turned out to be potent inducers of apoptosis in Jurkat Vector cells inducing cell death in a concentration- and time-dependent manner in the μM dose range (Figs. 14 and 15 as well as data not shown). Because of the known proapoptotic effects of sanguinarine and chelerythrine, only apoptosis inducing potency of chelidonine, protopine and allocryptopine was analysed in more detail. In these investigations chelidonine turned out to be a potent inducer of apoptosis in Jurkat Vector cells inducing cell death in a concentration- and time-dependent manner (Fig. 14a and data not shown). 1 μM chelidonine was sufficient to induce apoptosis in Jurkat Vector and A3 cells within 24 h as determined by flow cytometry (Fig. 14a left panel, white bars). Maximal effects were almost obtained at a concentration of 5 μM and a further increase in the chelidonine-concentration did not result in a further massive increase in apoptosis rates. Flow cytometric data revealed strong chelidonine-induced mitochondrial damage and caspase-activation in the same dose range (Fig. 14a left panel grey and black bars, respectively). Micrographs showing typical apoptotic nuclear morphology of Hoechst 33342-stained cells are depicted in Fig. 14a (right panel) Interestingly, even at increased chelidonine concentrations, only moderate levels of necrotic cell death (below 20%) were observed (data not shown).

The alkaloid protopine (≥ 50 μM) also exerted cytotoxic effects on Jurkat T-lymphoma cells. However, increased drug concentrations compared to chelidonine were required to induce similar levels of cell death (approximately 40% of cell death upon treatment with 50 μM protopine compared to 5 μM chelidonine) (Fig. 14b). Interestingly, fluorescence microscopy of protopine-treated cells upon combined staining with Hoechst 33342 and propidium iodide revealed that a further increase in the alkaloid concentration led to increasing rates of necrotic cell death (data not shown).

In contrast to the above mentioned findings the alkaloid allocryptopine had only minor apoptosis inducing potency. At concentrations between 50 and 150 μM the drug almost completely failed to induce mitochondrial damage, caspase-activation, chromatin condensation or nuclear fragmentation (Fig. 15, left panel). Only at increased drug concentrations (250 μM to 1 mM) allocryptopine induced cell death including apoptosis and necrosis (Fig. 15, right panel).

**Figure 16:**
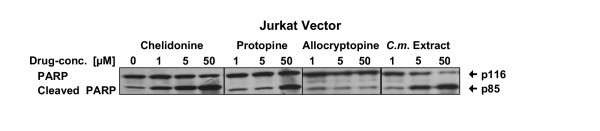
***Chelidonium majus *L. alkaloids induce PARP-cleavage**. Jurkat Vector cells were treated for 24 h with medium or 1, 5 and 50 μM chelidonine, protopine, allocryptopine, and *Chelidonium majus *extract adjusted to 1, 5 or 50 μM chelidonine. Caspase-activation was then determined by Western blot analysis with antibodies specific for PARP and cleaved PARP. A representative experiment is shown.

These data were further substantiated by determination of cleavage of the caspase-3 substrate PARP. In this regard, Western blot analysis of cytosolic extracts upon treatment of Jurkat Vector cells with chelidonine, protopine, allocryptopine and *Chelidonium majus *L. extract revealed strong caspase-activation indicated by massive PARP-cleavage only upon treatment with 1 μM chelidonine, 50 μM protopine and 5 μM *Chelidonium majus *L. extract, respectively, while in the dose range tested, allocryptopine failed to induce PARP-cleavage (Fig. 16).

### Influence of Bcl-2 on chelidonine-induced apoptosis

**Figure 17:**
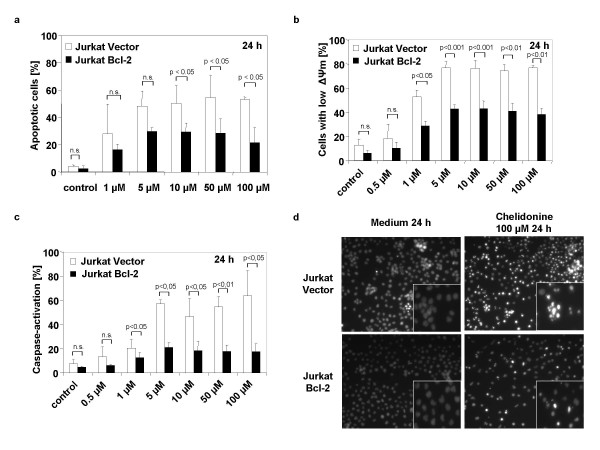
Chelidonine-induced apoptosis is partially inhibited by anti-apoptotic Bcl-2/b>: Jurkat Vector and Bcl-2 overexpressing cells were treated for 24 h with medium supplemented with solvent or 1, 5, 10, 50 and 100 μM chelidonine. Apoptosis induction was then quantified using **(a) **light scatter characteristics to determine cellular shrinkage, **(b) **TMRE-staining to detect alterations of mitochondrial membrane potential Δψm, and **(c) **CaspACE^®^-staining to test caspase-activation. Data show means ± SD (n = 3). Paired t-test was performed using GraphPad InStat version 3.00 for Windows 95, GraphPad Software, San Diego California USA, . **(d) **Combined staining with Hoechst33342 and propidium iodide of Jurkat Vector cells treated for 24 h with medium or 100 μM chelidonine revealed massive apoptosis while apoptosis rates were reduced in Bcl-2 overexpressing Jurkat cells.

Upon definition of chelidonine as a potent apoptosis inducing component of Ukrain and *Chelidonium majus *L. extracts, we wondered whether Bcl-2 would also interfere with apoptosis regulation of this isolated alkaloid component. As expected, Bcl-2 clearly reduced chelidonine-induced mitochondrial damage, caspase-activation and apoptosis at all concentrations tested, arguing for an involvement of Bcl-2 in the regulation of chelidonine-induced apoptosis (Fig. 17a–d).

## Discussion

Our data clearly show that the antineoplastic drug preparation Ukrain (NSC-631570) is a potent inducer of apoptosis and cell death in human Jurkat T lymphoma cells involving mitochondrial damage and caspase-activation. However, our data also reveal that the cytotoxic efficacy of Ukrain is not due to the suggested "Ukrain-molecule" but instead to apoptosis inducing alkaloids from *Chelidonium majus *L..

It has been shown earlier that Ukrain induces apoptosis in tumour cell lines from human solid tumours [9,10,12,13]. Here, we show for the first time that Ukrain is also effective in a Jurkat T-Lymphoma cell model. Interestingly, the concentrations required for efficient induction of apoptosis in Jurkat cells (1–50 μg/ml) were similar to the concentration required for the reported radiosensitizing effects of Ukrain on human colorectal and glioblastoma cell lines *in vitro *(0.1–50 μg/ml) [6].

However, up to now the mechanism of Ukrain-induced apoptosis remained unclear. In our hands, apoptosis induction did not require the presence of caspase-8 and FADD, two integral signalling molecules of the death receptor pathway. In line with these findings, expression of the caspase-8 inhibitor cFLIP-L or resistance to the death receptor ligands CD95 and TRAIL failed to abrogate Ukrain-induced apoptosis. These findings implicate that Ukrain induces apoptosis independently from death receptor signalling. The delayed activation of caspase-3 and cleavage of the caspase-3 substrate PARP in the FADD-deficient cells could be indicative for the contribution of a FADD-dependent signalling event.

In contrast, overexpression of Bcl-2, Bcl-x_L _and expression of a dominant negative caspase-9 reduced Ukrain-induced apoptosis revealing an involvement of the mitochondrial signalling cascade that is controlled by anti-apoptotic members of the Bcl-2 family. A similar protective effect of Bcl-2 on Ukrain-induced apoptosis has also been reported for human keratinocytes [41]. However, the moderate inhibitory action of Bcl-2, Bcl-x_L _and caspase-9 DN on Ukrain-induced mitochondrial damage and caspase-activation suggests that some factors different from Bcl-2, Bcl-x_L _and caspase-9 may be required for regulation of mitochondrial damage and apoptosis execution upon Ukrain-treatment. In this regard, our data reveal that altered expression levels or integrity of the proapoptotic Bcl-2 family members Bak, Bid or Bad were not involved in Ukrain-induced apoptosis.

Our findings on the composition of the pharmaceutical preparation implicate that several distinct alkaloid compounds contribute to the proapoptotic signalling of Ukrain. In this regard, mass spectrometric analyses of the pharmaceutical preparation identified several well-known alkaloids from *Chelidonium majus *L. including chelidonine, chelerythrine, sanguinarine, protopine and allocryptopine as major constituents of the drug preparation without evidence for the presence of the postulated "Ukrain-molecule" or some dimeric or monomeric condensation products of thiotepa with two or one chelidonine moieties.

Interestingly, the same alkaloids were detected in a commercially available *Chelidonium majus *L. extract. Of these, chelerythrine, sanguinarine, chelidonine and to a lesser extent protopine turned out to potently induce apoptosis in Jurkat T-lymphoma cells, while allocryptopine was almost ineffective. Together with the observation that extracts from *Chelidonium majus *L. induced apoptosis of Jurkat T-lymphoma cells in a similar dose range as the reference alkaloid chelidonine and Ukrain these findings implicate that the pharmaceutical preparation Ukrain may mainly constitute purified extracts from *Chelidonium majus L*. and that the *Chelidonium majus *L. alkaloids chelidonine, chelerythrine, sanguinarine and protopine are responsible for the reported cytotoxic effects of the drug preparation. Our findings are consistent with a recent publication from Panzer and co-workers that similarly failed to confirm the presence of the specified "Ukrain-molecule" by thin-layer chromatography, high-performance liquid chromatography and liquid chromatography-mass spectrometry and gave raise to the assumption that Ukrain preparations may consist of a mixture of several *Chelidonium majus *L. alkaloids [35].

*Chelidonium majus *L. and its alkaloids are well-known to exert antitumour, antiinflammatory and antimicrobial effects and to affect diverse cellular processes [42-47]. It has been shown that chelerythrine, sanguinarine and chelidonine exert cytotstatic and cytotoxic effects on human tumour cell lines [48-54]. In this context, the common antimicrotubule properties of chelidonine, chelerythrine and sanguinarine have been suggested to contribute to their observed antineoplastic activity [49,55]. Moreover, chelerythrine was found to function as an antagonist of antiapoptotic Bcl-x_L _and to inhibit, similar to its structural homologue sanguinarine, the anti-apoptotic mitogen-activated protein kinase phosphatase as well as protein kinase C [52,56-59].

Up to now only single reports revealed growth inhibitory and pro-apoptotic effects of chelidonine [49,51]. In the present investigation, we identified chelidonine as a potent proapoptotic component of Ukrain and *Chelidonium majus *L. extract. Thus, in addition to chelerythrine and sanguinarine chelidonine may contribute to the reported cytotoxic efficacy of both preparations. Interestingly, while protopine, chelerythrine and sanguinarine induced high levels apoptosis and necrosis, the predominant mode of cell death upon chelidonine-treatment was apoptosis. This is consistent with recent data of Kemeny-Beke and co-workers that observed similar effects in a primary human uveal melanoma cell line [51].

Moreover, we show for the first time that apoptosis induction by chelidonine involves alteration of mitochondrial function and caspase-activation. The observation that overexpression of Bcl-2 interfered with chelidonine-induced apoptosis argues for a role of the intrinsic mitochondrial pathway. These data are reminiscent to recent findings revealing the involvement of the mitochondrial death pathway in sanguinarine-induced apoptosis in immortalised keratinocytes [60]. However, Bcl-2 failed to completely inhibit chelidonine-induced proapoptotic effects pointing to the contribution of Bcl-2-independent signalling events.

Our data implicate that defined proapoptotic alkaloid compounds from *Chelidonium majus *L. may constitute promising novel anticancer drugs [51]. In contrast, despite numerous reports on beneficial effects of Ukrain in preclinical investigations, case reports and non-randomized as well as randomized clinical trials, up to now there is no solid scientific basis for a rational use of Ukrain in cancer treatment. In this regard, the suggested selective antitumour activity of Ukrain is still controversial [7]. Moreover, a recent systematic review of published clinical trial data revealed that most of the clinical data do not meet stringent criteria for randomized clinical studies and that methodological limitations of the remaining studies (e.g. small sample size, poor or unknown method of randomisation) actually prevent final conclusions on the putative value of Ukrain for the treatment of cancer patients [8]. Our finding that Ukrain represents a mixture of *Chelidonium majus *L. alkaloids instead of the "Ukrain-molecule" further challenges clinical use of Ukrain and in addition rises concerns on potential adverse side effects of the drug preparation on liver tissue as observed for *Chelidonium majus *L. extracts [61,62].

## Conclusion

Taken together, our investigation clearly shows that induction of apoptosis contributes to the cytotoxic effects of the pharmaceutical preparation Ukrain. Ukrain induced apoptosis independently from death receptor signalling via a pathway involving mitochondrial damage and caspase-activation that were partially sensitive to overexpression of Bcl-2, Bcl-x_L _and a dominant negative caspase-9.

Importantly, our mass spectrometric data reveal that instead of the composition indicated by the manufacturer the pharmaceutical preparation Ukrain constitutes a mixture of *Chelidonium majus *L. alkaloids including chelidonine, protopine, allocryptopine, chelerythrine and sanguinarine. In addition to native *Chelidonium majus *L. extract the isolated alkaloids sanguinarine, chelerythrine and chelidonine were identified as potent inducers of apoptosis in Jurkat T-Lymphoma cells that induced cell death with similar potency as Ukrain. Consequently, our data strongly suggest that the observed antineoplastic effects of Ukrain are based on proapoptotic *Chelidonium majus *L. alkaloids instead of the hypothetic "Ukrain-molecule" and implicate that purified *Chelidonium majus *L. alkaloids should be considered for further preclinical and clinical development as single drugs or in combination with standard therapies including ionizing radiation.

## List of abbreviations

cFLIP, cellular FADD-like interleukin-1 converting enzyme (FLICE) inhibitory protein; FADD, Fas-associated death domain; PARP, poly(ADP-ribose)polymerase

## Competing interests

Ukrain and financial support were obtained from Nowicky Pharma.

## Authors' contributions

**Daniel Habermehl**: substantial contribution to the data acquisition, data analysis and drafting the manuscript.

**Bernd Kammerer**: substantial contribution to the acquisition and analysis and interpretation of of the data and drafting the manuscript.

**René Handrick**: substantial contribution to the analysis of the data, drafting the manuscript and critical revision.

**Therese Eldh**: substantial contribution to the acquisition of the data.

**Charlotte Gruber**: substantial contribution to the acquisition of the data.

**Peter Daniel: **substantial contribution to data interpretation and revision of the manuscript.

**Nils Cordes: **critical revision of the manuscript.

**Ludwig Plasswilm**: contribution to conception of the study and critical revision of the manuscript.

**Michael Bamberg**: critical revision of the manuscript.

**Claus Belka**: substantial contribution to the conception of the study, interpretation of data, critical revision of the manuscript and final approval.

**Verena Jendrossek: **substantial contribution to the conception and design of the study, interpretation of data, drafting of the manuscript, critical revision of the manuscript and final approval.

## Pre-publication history

The pre-publication history for this paper can be accessed here:
